# *Listeria monocytogenes* in Traditional Ready‐to‐Eat Dry Meat Products From Zagreb, Croatia: Occurrence and Genotyping

**DOI:** 10.1155/ijm/1946018

**Published:** 2026-07-10

**Authors:** Dora Tomašković, Lucija Hlebić, Lovran Peinović, Andrea Humski, Irena Reil, Maja Dopuđ, Sanja Duvnjak

**Affiliations:** ^1^ Laboratory for Food Microbiology, Department for Veterinary Public Health, Croatian Veterinary Institute, Zagreb, Croatia, veinst.hr; ^2^ Laboratory for Bacterial Zoonoses and Molecular Diagnostic of Bacterial Diseases, Department for Bacteriology, Croatian Veterinary Institute, Zagreb, Croatia, veinst.hr

**Keywords:** clonal complexes, food safety, *Listeria monocytogenes*, ready-to-eat meat products, traditional Croatian food, whole-genome sequencing

## Abstract

Contamination of ready‐to‐eat (RTE) foods with *Listeria monocytogenes* represents a significant public health concern, particularly due to the limited data available on its prevalence in traditional Croatian RTE products. This study is aimed at determining the prevalence of *L. monocytogenes* in traditional dried RTE meat products sampled from local markets in Zagreb, characterizing the genomic features of the isolated strains, and assessing their potential public health implications. A total of 50 RTE meat products, including Slavonian sausage, dry meat products, and garlic sausage, were collected and analyzed using standard cultural methods, followed by biochemical confirmation and serogroup identification with multiplex PCR. Whole‐genome sequencing (WGS) was employed to determine clonal complexes (CCs), sequence types (STs), and the presence of virulence‐associated genes. *L. monocytogenes* was detected in 22% (11/50) of the analyzed samples, of which 63.63% (7/11) were isolated from Slavonian sausage. Among the confirmed isolates, Serogroup IIa was the most prevalent (54.54%). WGS analysis revealed the presence of seven distinct CCs, of which CC9 (*n* = 3), CC37 (*n* = 2) and CC121 (*n* = 2) were the most common. One of the strains was identified as CC1, which is considered hypervirulent. Single‐nucleotide polymorphism (SNP) analysis demonstrated that two strains identified as CC121 and two strains identified as CC9 were highly related (seven SNP differences in each). Overall, these findings provide new insights into the presence of *L. monocytogenes* in traditional RTE meat products in Zagreb and highlight a potential health risk for consumers.

## 1. Introduction

*Listeria monocytogenes* (*L. monocytogenes*) is a ubiquitous foodborne pathogen capable of causing severe invasive infections, collectively known as listeriosis, in both humans and animals. Although sporadic, outbreaks of listeriosis are of particular concern due to the relatively high mortality rate compared with other foodborne illnesses [1, 2]. According to the European Food Safety Authority (EFSA) and the European Centre for Disease Prevention and Control (ECDC), listeriosis was the fifth most commonly reported zoonosis in Europe in 2023 and 2024, with a hospitalization rate of 96.5% and a mortality rate of 19.7% within the European Union [1, 2]. The disease poses the greatest risk to vulnerable populations, including the elderly, pregnant women, newborns, and immunocompromised individuals [3, 4]. Ready‐to‐eat (RTE) foods are considered a significant vehicle for *L. monocytogenes* transmission, particularly in Europe, where multiple risk assessments have highlighted these products as a major concern for consumers [5]. Several documented outbreaks have linked *L. monocytogenes* contamination to RTE meat products such as sausage salad (Germany), jellied pork (France and Austria), and stuffed pork (Spain) [6–9].

The samples analyzed in this study represent traditional Croatian artisanal dried meat products. The production of these products is deeply rooted in rural traditions and commonly sold at urban markets in Zagreb. Zagreb city markets serve as a platform where small‐scale producers bring their handcrafted sausages directly to consumers. Furthermore, these markets help preserve tradition by supporting small local economies and keeping artisanal food culture alive. Artisan dried meat products are widely consumed and produced using traditional methods, often without fully standardised processing conditions or consistent Hazard Analysis and Critical Control Point (HACCP) systems, which may increase the risk of microbiological contamination. Additional risks include manual handling, use of natural casings, and potential cross‐contamination during production and distribution [1].

As *L. monocytogenes* is widely distributed in the environment and frequently found in food processing, the production of food, especially RTE products, can be a challenge for food business operators (FBOs) [10, 11]. *L. monocytogenes* can enter the food processing environment either through incoming raw materials or through the movement of personnel and equipment and colonise food processing equipment and food contact surfaces. The bacterium can also form biofilms and persist on surfaces, leading to contamination of food products [12]. Food manufacturers are obliged to carry out their controls to monitor and control possible contamination with *L. monocytogenes* in the food processing environment and in the final product [13]. Therefore, Commission Regulation (EC) No 2073/2005 [14] established microbiological criteria for foodstuffs, requiring the absence of *L. monocytogenes* in 25 g or < 100 CFU/g at the end of shelf life, depending on the product category.

Given the public health implications, molecular characterization of *L. monocytogenes* is essential. Molecular serogrouping classifies isolates into major serogroups (IIa, IIb, IIc, IVa, IVb), with most human cases associated with Serogroups IVb, IIa, and IIb [15, 16]. In addition, clonal complex (CC) analysis provides insight into strain virulence and epidemiology, allowing classification of isolates as infection‐associated, food‐associated, or intermediate based on their source prevalence. Clones are categorized as “infection‐associated,” “food‐associated,” or “intermediate” based on their source prevalence. The “infection‐associated” CCs (e.g., CC1, CC2, CC4, and CC6) are often associated with central nervous system (CNS) infections, neonatal–maternal (NM) infections, and bacteraemia [17, 18]. In contrast, “food‐associated” CCs (e.g., CC121 and CC9) are more frequently isolated from food and tend to cause disease in immunocompromised populations [19–21]. The “infection‐associated” clones are considered hypervirulent due to the incidence of disease, whereas the “food‐associated” strains are less invasive and considered hypovirulent. Virulence is further influenced by pathogenicity islands such as LIPI‐1, which is essential for intracellular survival and spread, as well as internalins involved in host cell invasion [22, 23].

Whole‐genome sequencing (WGS) was used in the present study to characterize *L. monocytogenes* isolates at the genomic level. WGS enables high‐resolution typing of strains, including assignment to sequence types (STs) and CCs, as well as single‐nucleotide polymorphism (SNP) analysis for detailed comparison of isolates in molecular epidemiological investigations [24, 25]. Although SNP‐based approaches provide high discriminatory power, they can be difficult to standardise and interpret [26].

Currently, there is limited data on the diversity and genomic characteristics of *L. monocytogenes* isolated from traditional RTE food products in Croatia. The distribution of CCs and the presence of virulence genes in strains derived from traditional dry meat RTE products remain largely undocumented, making it difficult to assess the potential public health risk posed by these products. Therefore, the aim of this study was to characterize *L. monocytogenes* strains isolated from traditional Croatian dried meat products using multiplex PCR and WGS, with a focus on their pathogenic potential and public health relevance.

## 2. Materials and Methods

### 2.1. Sampling

The samples were taken from eight city markets at different locations in Zagreb, Croatia (Dolac, Kvaternik Square, Dubrava, Trešnjevka, Branimir Square, Borongaj, Jarun, and Utrine). A total of 50 samples were collected for the detection and enumeration of *L. monocytogenes.* Sampling was conducted over a period of approximately 10 months at selected urban market locations specializing in traditional artisanal products. These locations were chosen due to their high consumer turnover and relevance for public exposure. All samples were traditional Croatian RTE dried meat products. The sampled products originated from multiple producers, with some obtained from the same producer and others from different manufacturers.

### 2.2. Analysis of Samples

Samples were analyzed according to the method ISO 11290‐1:2017 [27] for the detection of *L. monocytogenes* and ISO 11290‐2:2017 [28] for quantitative analysis (CFU/g). In brief, for the detection of *L. monocytogenes*, 25 g of each sample was cultured in 225 mL of half Fraser enrichment broth (Merck, Germany) for 25 ± 1 h at 30^°^C ± 1^°^C. After incubation, plating was performed on two selective agar plates: agar *Listeria* according to Ottavani and Agosti—ALOA (Merck, Germany) and Oxford (Merck, Germany)—which were incubated for 24–48 h at 37°C. In addition, 0.1 mL of broth was cultured in 10 mL of Fraser broth (Merck, Germany), incubated for 24 h at 37°C and plated on two selective agar plates (ALOA and Oxford). The plates were analyzed for typical *L. monocytogenes* colonies (blue/green with halo) and streaked on Tryptic Soy Agar with yeast extract (TSA‐YE) (Merck, Germany) and blood agar (Oxoid, UK) incubated for 24–48 h at 37°C. Suspect colonies were confirmed as *L. monocytogenes* by morphology, haemolysis, catalase activity, and biochemical tests (D‐xylose and L‐rhamnose). The Vitek2 system (bioMérieux, France) was used as a confirmatory method to verify the identity of presumptive *L. monocytogenes* isolates prior to further molecular analyses. The strain *L. monocytogenes* ATCC 13932 was used as quality control. After confirmation, the strains were stored in TSB with 15% glycerol at −80°C.

Samples were analyzed for the detection and enumeration of *L. monocytogenes.* In the case of suspected colonies, samples were also subjected to quantitative analysis, in which 10 g of each sample was mixed with 90 mL of buffered peptone water (Merck, Germany) and plated on ALOA agar. The plates were then incubated for 24–48 h at 37°C, with a final result of < 10 CFU/g.

### 2.3. Genomic DNA Extraction

Bacterial isolates were grown on blood agar (Oxoid, United Kingdom) and incubated for 24–48 h at 37°C. DNA was extracted using the NucleoSpin Microbial DNA Kit (Macherey‐Nagel, Germany) by mechanical disruption at a frequency of 30 Hz for 30 min according to the manufacturer′s protocol. The quality and concentration of extracted DNA were assessed using a DS‐11 spectrophotometer (DeNovix, United States) and a Qubit 4 fluorometer (Invitrogen, United States).

### 2.4. Serogrouping

Serogrouping was performed using multiplex PCR [16]. Amplification reactions were performed using a Proflex PCR device (Applied Biosystems, United States). This method enables the differentiation of 13 different serotypes, which are divided into five serogroups: IIa (1/2a and 3a), IIb (1/2b, 3b, and 7), IIc (1/2c and 3c), IVa (4a and 4c), IVb (4b, 4d, and 4e), and non‐*L. monocytogenes* species [16]. *L. monocytogenes* isolates were successfully serogrouped by amplification of five target genes: *prs*, *lmo0737*, *lmo1118*, *orf2110*, and *orf2819* [16]. Primers used for molecular serotyping are from Doumith et al. [29], which is a part of the ANSES protocol. All primers were used at a concentration of 0.2 *μ*M, except *lmo1118*, which was used at 0.4 *μ*M. Primer concentrations were optimised based on preliminary experiments to ensure robust amplification. Primer sequences and concentrations are listed in Table S1.

Multiplex PCR was performed using the Multiplex PCR Kit (QIAGEN, Germany) with a reaction volume of 20 *μ*L. The mixture consisted of 10 *μ*L of 5x PCR master mix, 4 *μ*L of nuclease‐free water, 4 *μ*L of 5x primer mix, and 2 *μ*L of DNA (0.5 ng/*μ*L) extracted from bacterial strains of *L. monocytogenes*. Positive and negative controls were used in each run to validate the results. The positive control contained known *L. monocytogenes* DNA to confirm the amplification process, whereas the negative control used nuclease‐free water instead of DNA to detect possible contamination. The thermal cycling program for multiplex PCR included an initial denaturation step at 95°C for 15 min, followed by 35 cycles at 94°C for 30 s, 53°C for 40 s, and 72°C for 90 s. A final extension step was performed at 72°C for 7 min.

The PCR products were analyzed using the capillary electrophoresis system QSep100 (Nippon Genetics, Japan). The system provided electropherograms with accurate fragment size determination, allowing confirmation of target gene amplification according to the reference [16]. PCR‐based serogrouping was used as a complementary method to confirm isolate classification and to ensure agreement with WGS‐derived data.

### 2.5. WGS

The library for Illumina sequencing was prepared following the manufacturer′s instructions for the Illumina DNA Prep kit (Illumina, United States). The sequencing was performed on a MiSeq device (Illumina, United States) using a MiSeq Reagent Kit v2.

Raw sequencing reads were trimmed using Trimmomatic [30], and the filtered sequences were aligned using SPAdes [31]. The quality of the assembly was assessed using QUAST [32]. Raw sequence files National Library of Medicine (NCBI) under Accession Number PRJNA1331686, Submission ID: SUB15644684 (http://www.ncbi.nlm.nih.gov/bioproject/1331686).

### 2.6. CC and ST

Multilocus sequence typing (MLST) analysis was performed by extracting the alleles of seven housekeeping genes *abcZ*, *bglA*, *cat*, *dapE*, *dat*, *ldh*, and *lhkA* from assembled genomes [33]. These alleles were compared with profiles from the Institut Pasteur MLST *Listeria* database [34] to assign STs and determine CCs.

### 2.7. Virulence Genes Detection

Virulence genes were identified using the Virulence Factors Database (VFDB) [35], according to previous studies [36].

### 2.8. SNPs Analysis

SNPs were identified using the CSI Phylogeny 1.4 pipeline available on the Center for Genomic Epidemiology, on fastq files, and with default parameters [37]. The generated Newick file was visualized using MEGA Version 12.0.14 [17]. The tree is drawn to scale, with branch lengths measured in the number of substitutions per site.

## 3. Results

### 3.1. Occurrence of *L. monocytogenes*

A total of 50 samples of traditional dried meat products obtained from eight city markets in Zagreb were analyzed. All samples were classified as traditional RTE foods. The overall occurrence of *L. monocytogenes* across the sampled markets is presented in Table 1, with the pathogen confirmed in 11 samples (22%).

**Table 1:** Results of detection and enumeration of *L. monocytogenes* in Zagreb city markets.

City market	Number of samples	Detection (positive in 25 g)	Enumeration (CFU/g)
Dolac	9	2	< 10
Kvaternik square	7	1	< 10
Dubrava	6	1	< 10
Trešnjevka	6	1	< 10
Branimir square	3	1	< 10
Borongaj	1	—	< 10
Jarun	7	3	< 10
Utrine	11	2	< 10
Total	50	11	< 10

Of the positive samples, seven isolates (63.63%; *n* = 7) originated from Slavonian sausage, two (18.18%; *n* = 2) from garlic sausage, one (9.09%; *n* = 1) from dried meat products (dry neck), and one (9.09%; *n* = 1) from home‐made fermented sausage. A detailed distribution of positive samples by product type and market is shown in Table 2.

**Table 2:** Detailed distribution of *L. monocytogenes*–positive samples by product type and market.

City market	Sample type	Number of positive samples
Dolac	Game sausage (2), Samobor sausage (1), Slavonian sausage (2), dry ham (1), dry smoked sausage (1), and dry meat product (dry neck) (1)	Slavonian sausage (LM08) and dry meat product (dry neck) (LM09)
Kvaternik square	Dry meat product (dry neck) (2), dry meat product (2), Slavonian sausage (2), and dry sausage (1)	Slavonian sausage (LM17)
Dubrava	Garlic sausage (2), Slavonian sausage (1), homemade sausage (1), dry meat product (1), and Srijem sausage (1)	Garlic sausage (LM22)
Trešnjevka	Garlic sausage (1), dry smoked sausage (1), Slavonian sausage (1), kulen‐style dry sausage (1), and homemade spicy sausage (2)	Garlic sausage (LM24)
Branimir square	Kulen‐style dry sausage (1), Slavonian sausage (1), and homemade sausage (1)	Slavonian sausage (LM31)
Borongaj	Homemade sausage (1)	0
Jarun	Slavonian sausage (4), game sausage (1), homemade sausage (1), and Vrbovec sausage (1)	Slavonian sausage (LM35, 36, 38)
Utrine	Slavonian sausage (5), garlic sausage (1), homemade sausage (2), game sausage (1), dry meat product (dry neck) (1), and Baranjska sausage (1)	Slavonian sausage (LM43) and homemade sausage (LM44)
Total	50	11

All positive samples were further analyzed using a quantitative method, with bacterial counts below the limit of quantification (< 10 CFU/g).

### 3.2. Serogrouping

Although serotyping can be inferred from WGS data, conventional serogrouping was performed as part of routine laboratory characterization and to enable comparison with previous studies and historical surveillance data. Serogroup analysis revealed that of the 11 confirmed *L. monocytogenes* strains, six (54.54%; *n* = 6) were assigned to Serogroup IIa, followed by three strains assigned to Serogroup IIc (27.27%; *n* = 3), one strain (9.09%; *n* = 1) to Serogroup IIb, and one strain (9.09%; *n* = 1) to Serogroup IVb (Table 3). The results of serogroup PCR are shown in Figure S1.

**Table 3:** Distribution of *L. monocytogenes* strains according to serogroup, clonal complex, and sample.

Strain	Strain identification	Serogroup	Clonal complex	Sample
1	LM08	IIa	CC37	Slavonian sausage
2	LM09	IIa	CC8	Dry meat product (dry neck)
3	LM17	IIa	CC199	Slavonian sausage
4	LM22	IIb	CC5	Garlic sausage
5	LM24	IIa	CC121	Garlic sausage
6	LM31	IVb	CC1	Slavonian sausage
7	LM35	IIa	CC121	Slavonian sausage
8	LM36	IIc	CC9	Slavonian sausage
9	LM38	IIc	CC9	Slavonian sausage
10	LM43	IIc	CC9	Slavonian sausage
11	LM44	IIa	CC37	Homemade sausage

### 3.3. Distribution of *L. monocytogenes* Clones (CC) and STs

A total of 11 strains were classified into seven CCs (Table 3). The distribution of CCs showed that CC9 was the most prevalent (27.27%; *n* = 3), followed by CC37 (18.18%; *n* = 2) and CC121 (18.18%; *n* = 2), whereas CC8, CC199, CC5, and CC1 were each represented by a single isolate: CC8 (9.09%; *n* = 1), CC199 (9.09%; *n* = 1), CC5 (9.09%; *n* = 1), and CC1 (9.09%; *n* = 1). Of the six strains identified as IIa, two were assigned to CC37 (33.33%; *n* = 2), two to CC121 (33.33%; *n* = 2), one to CC199 (16.66%; *n* = 1), and one to CC8 (16.66%; *n* = 1). All strains defined as Serogroup IIc were assigned to CC9 (*n* = 3). One strain, defined as Serogroup IIb, was assigned to CC5 (*n* = 1), and one strain, designated as IVb, was assigned to CC1 (*n* = 1) (Table 3).

The STs corresponded to the specified CCs. The strain identified as ST9 was determined to be CC9, followed by ST37 (CC37), ST121 (CC121), ST8 (CC8), ST199 (CC199), ST5 (CC5), and ST1 (CC1).

### 3.4. Determination of Virulence Genes

**Figure 1:**
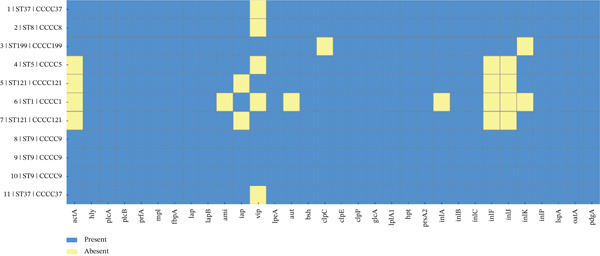
Heatmap showing the presence and absence of virulence genes among the analyzed *Listeria monocytogenes* strains. Among the 11 analyzed strains, CC9 was the most prevalent clonal complex (27.27%; *n* = 3), followed by CC37 (18.18%; *n* = 2) and CC121 (18.18%; *n* = 2). Other clonal complexes, including CC8, CC199, CC5, and CC1, were each represented by a single strain (9.09%; *n* = 1).

WGS data were analyzed for the presence of virulence genes. All isolates harbored the *Listeria* Pathogenicity Island 1 (LIPI‐1); however, the *actA* gene was absent in isolates belonging to CC1, CC121, and CC5. Consequently, a complete LIPI‐1 gene set was identified in 63.64% (*n* = 7) of the isolates, whereas incomplete LIPI‐1 profiles, lacking *actA*, were observed in 36.36% (*n* = 4), including the hypervirulent CC1 strain. In addition to LIPI‐1, the presence of other *Listeria* pathogenicity islands was assessed. LIPI‐2–associated genes were detected, whereas no virulence genes related to LIPI‐3 or LIPI‐4 were identified in the analyzed isolates. All isolates tested positive for key virulence factors involved in bile resistance (*bsh*), intracellular survival (*lplA1*, *hpt*, and *prsA2*), surface protein anchoring (*lspA*), and peptidoglycan modification (*oatA* and *pdgA*). Among the four adherence genes assessed, *fbpA*, *lap*, and *lapB* were present in all isolates, whereas *ami* was absent only in the CC1 isolate. This CC1 isolate also lacked *gtcA*, which is involved in teichoic acid biosynthesis, and *aut*, an invasion‐related gene, both of which were found in all other strains. Internalin genes were broadly distributed, with *inlB*, *inlC*, and *inlP* detected in all isolates. Notably, *inlA*, which plays a key role in epithelial cell invasion, was absent in the CC1 isolate. In contrast, *inlF* and *inlJ* were present in CC37, CC8, CC199, and CC9, whereas *inlK* was detected in all isolates except CC1 and CC199. Regarding stress response genes, *clpCEP* was detected in all isolates, whereas *clpC* was missing in CC199. The invasion‐associated gene *ipeA* was present across all isolates. Interestingly, *vip*, a gene implicated in immune evasion, was found in CC199, CC121, and CC9, whereas *cwhA* was absent in CC121. These results suggest notable differences in virulence gene profiles across CCs, with food‐adapted CCs (e.g., CC9 and CC121) showing stress adaptation traits. At the same time, the hypervirulent CC1 displayed an unexpectedly reduced virulence gene complement. Complete gene distributions are presented in Figure 1.

### 3.5. SNPs Analysis

**Figure 2:**
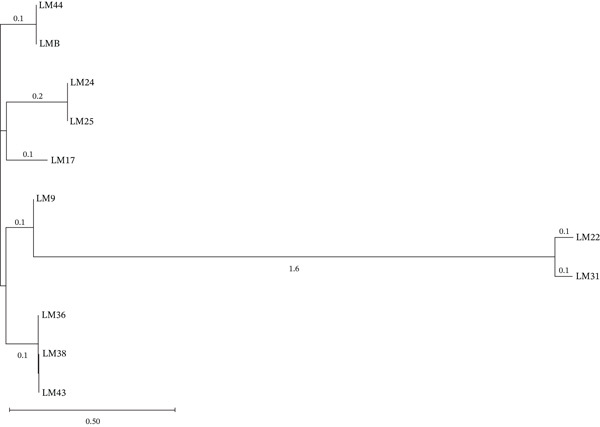
Phylogenetic relationship among *L. monocytogenes* strains based on single‐nucleotide polymorphism (SNP) analysis. The phylogenetic tree was generated using the maximum likelihood method. Branch lengths represent the number of substitutions per site. Closely related strains LM24/LM35 and LM38/LM43 differed by seven SNPs, whereas LM44 and LM08 differed by 44 SNPs.

All of the isolated strains were subjected to SNP analysis to assess whether any of them may be closely linked. To obtain a broad overview of the SNP profiles, each isolate was initially compared with a reference strain of the same serotype. A maximum likelihood tree was generated for each comparison showing the relationship between the isolates. The SNP tree clustered the *L. monocytogenes* isolates according to their ST/CC (Figure 2). Genomic relatedness between isolates was assessed based on SNP differences, as described previously [25] (Table S2). Among the analyzed strains, LM38 and LM43 (CC9/ST9) as well as LM24 and LM35 (CC121/ST121) displayed high genetic similarity, differing by only seven SNPs. Strains LM44 and LM08, identified as CC37/ST37, displayed a higher level of genetic variation, differing by 44 SNPs. The other strains showed a high SNP distance and are therefore considered genetically unrelated.

## 4. Discussion

This study presents the detection and molecular characterization of *L. monocytogenes* strains isolated from traditional dried RTE meat products collected from retail markets in Zagreb, Croatia. The pathogen was detected in 22% (11/50) of the analyzed samples. In contrast to these findings, previous Croatian studies reported the absence of *L. monocytogenes* in various traditional meat products, including homemade sausages made from rabbit, deer, roe deer, and wild boar [38], as well as in traditionally produced raw sausages [39]. However, these earlier studies were limited by small sample sizes—Markov et al. [38] analyzed only 15 samples, and Kozačinski et al. [39] focused on a single small production facility—which may have contributed to the absence of positive findings. Although the present study is also limited by a relatively small sample size (*n* = 50), the detection of *L. monocytogenes* in over one‐fifth of the tested products underscores the need for broader investigations. Future research should include more extensive sampling and microbiological testing of traditional RTE meat products to more accurately assess the prevalence, distribution, and potential public health risk posed by *L. monocytogenes*.

Supporting this need, Pažin et al. [40] identified the presence of L. monocytogenes at various points in the production process of fermented sausages, including on equipment, work surfaces, and within the sausages themselves during curing. Similarly, Kiš et al. [41] reported a lower contamination rate of 3.48% (8/230 samples) in RTE meat products. Furthermore, other studies have confirmed the presence of L. monocytogenes in nonmeat RTE foods such as salads [42] and cakes [43], highlighting the relevance of this pathogen across the food production sector. This is in agreement with recent findings from Romania, where molecular characterization of L. monocytogenes isolated from RTE food products also emphasized the importance of continuous surveillance to improve food safety and public health protection [44].

All positive samples in this study originated from traditional meat products sold at urban markets in Zagreb. These products are widely consumed in Croatia and are often produced under natural and nonstandardised conditions. Notably, *L. monocytogenes* was mainly isolated from Slavonian sausage, which is probably related to the traditional production process, often carried out under natural and uncontrolled conditions. The production of sausage is seasonal, and the quality of the product can be influenced by many factors such as raw material, recipe, technological parameters, and microlocation of production [45]. The detection of *L. monocytogenes* in Slavonian sausage and other products represents a public health concern, particularly for vulnerable population groups. All samples analyzed in this study came from retail outlets, to be exact from urban markets, which are popular places to buy traditional products in Croatia. The presence of *L. monocytogenes* was detected in samples from all markets in Zagreb included in the present study, except for the Borongaj market, represented with only one sample, which is a limitation for providing accurate data. These data are consistent with the latest EU zoonoses report, which indicates that the highest contamination rates of *L. monocytogenes* are observed in meat products, particularly fermented sausages, at the distribution stage [2]. Contamination of food can occur during the manufacturing process or through contaminated meat, as the presence of *L. monocytogenes* has been reported in animals, mainly in cattle, small ruminants, and pigs [2]. Based on these findings, the control of *L. monocytogenes* in RTE foods requires food manufacturers to implement effective control measures that include good hygiene and manufacturing practices during production and distribution.

All isolated strains in this study exhibited typical phenotypic characteristics, and subsequent genotyping provided valuable insights into their epidemiological significance.

Among the 11 characterized isolates, the most prevalent CCs were CC9 (27.27%), CC37 (18.18%), and CC121 (18.18%). Single isolates of CC1, CC5, CC8, and CC199 were also identified. These findings are consistent with the EFSA–ECDC joint typing report, which identifies CC9, CC121, and CC8 as among the most frequently detected clones in food isolates across the European Union [46]. Epidemiological data combined with genetic sequencing information and results from animal models indicate that 12 CCs make up almost 80% of all isolates [13]. In support of this, Félix et al. [47] analyzed a European dataset of 1484 *L. monocytogenes* genomes from 19 countries and diverse ecological niches.

Their findings demonstrated that food‐associated CCs such as CC9, CC121, CC8, CC37, and CC155 were predominantly isolated from RTE foods and food production environments, particularly in fish and meat products. In contrast, hypervirulent CCs, including CC1, CC2, CC4, and CC6, were mainly associated with animal clinical infections and animal‐derived samples. These results further emphasize the different distribution between CCs while highlighting the public health risk posed by hypervirulent clones more commonly found in invasive human listeriosis cases.

It is also important to mention that *L. monocytogenes* consists of at least four evolutionary lineages with the majority of isolates clustered into two lineages (I and II). Lineage I strains are usually isolated from human clinical cases, whereas Lineage II strains are commonly isolated from food and environmental sources [48]. Lineages I and II are strongly associated with specific CCs and STs. Lineage I predominantly includes hypervirulent CCs and STs such as ST1, ST2, ST5, ST6 and is more frequently associated with clinical cases, whereas Lineage II is mainly composed of food‐adapted CCs and STs, including ST7, ST8, ST9, ST37, commonly isolated from food and environmental sources [49]. Accordingly, the majority of isolates identified in this study (CC9, CC37, CC121, CC8, and CC199) belong to Lineage II, whereas the single CC1 isolate represents Lineage I.

Particularly noteworthy in this study is the identification of one CC1 isolate, a hypervirulent clone frequently associated with clinical listeriosis and classified within Serogroup IVb [50]. This serogroup is known to account for the majority of human infections [48]. Previous studies confirm CC1 as a major hypervirulent clone involved in CNS and NM infections, often in otherwise healthy individuals [21, 50, 51]. These data are consistent with the Joint Report on *L. monocytogenes* [46], which states that most human *L. monocytogenes* isolates are identified as CC1 and CC8. Similarly, CC8—also detected in this study—is associated with human listeriosis and has been linked to several significant outbreaks, including a multicountry outbreak in Europe [46] and the 2008 Canadian outbreak traced to RTE meat [52]. Despite its clinical relevance, CC8 is mainly associated with bacteraemia and, like CC9 and CC121, is more frequently isolated from food than clinical settings [21].

Strains isolated in this study were mostly classified as hypovirulent clones, such as CC121 and CC9. In contrast to CC1, these clones are primarily associated with bacteraemia and isolated from severely immunocompromised patients [21]. In addition, CC121 and CC9 are strongly associated with food and rarely cause disease in humans. Their importance in the food industry is emphasized due to the formation of biofilms [20, 21], which could explain their prevalence. Myintzaw et al. [53] also note that CC9 is one of the clones that has a higher proportion of biofilm, stress survival, and antimicrobial genes than other CC types. The latter also explains the high representation of CC9. In a study by Fagerlund et al. [54], CC121 is described as the most frequently detected CC in processing plants, followed by CC7, CC8, and CC9, with CC9 occurring mainly in meat processing plants. Therefore, the relatively higher representation of CC121 and CC9 observed in this study may be explained by the food matrix, as these clones are frequently associated with meat products [20]. In line with previous studies, CC9 and CC121 are commonly reported among food‐derived isolates, suggesting their adaptation to food processing environments [18, 47]. However, given the limited sample size, these findings should be interpreted with caution.

Other CCs identified in this study included CC5, which was detected in a single sample, and CC37, which was detected in two samples. Although CC5 is generally classified as hypovirulent [21], it has been associated with an outbreak in the United States in 2011 [55] and was considered critical in a recent study by Centorotola et al. [56]. CC37, more commonly associated with dairy products [57, 58], is also found in pig and ruminant farms and has been linked to animal listeriosis [59, 60]. Its public health significance remains unclear and requires further investigation.

Although the majority of isolates in this study were classified as hypovirulent, this does not exclude their pathogenic potential, particularly in immunocompromised individuals [61]. Fotopoulou et al. [50] highlighted that the highest proportion of human clinical isolates belonged to CC1; however, other CCs, such as CC9 and CC121—both detected in this study—have also been linked to human listeriosis, but at lower frequencies. It should be emphasized that virulence and disease‐causing potential can vary between individual strains, and that host susceptibility as well as food handling practices play a crucial role in the overall risk of foodborne illness [62, 63]. Furthermore, the prediction of human health risk in *L. monocytogenes* strains is also achieved by the detection of virulence genes. All isolates harbored the LIPI‐1, a core virulence element [23]; however, the *actA* gene was absent in four strains, including the CC1 isolate. As *actA* encodes a protein essential for actin‐based intracellular motility [64, 65], its absence may influence the efficiency of cell‐to‐cell spread and virulence expression, although it does not necessarily affect the ability to cause infection. In the present study, the primary focus on LIPI‐1 is justified by its central role in the pathogenicity of *L. monocytogenes*, whereas LIPI‐2 is mainly associated with *L. ivanovii* [66]. Nevertheless, internalin genes, which are a part of LIPI‐2, especially *inlA* and *inlB*, play a crucial role in epithelial invasion and virulence [67]. In this study, *inlB* was detected in all isolates, whereas *inlA* was missing only in the CC1 strain. Although this might suggest reduced invasiveness [68], Maury et al. [20] demonstrated that CC1 strains are well adapted for intestinal colonization, persistence, and transmission. For the strain identified as CC1, the absence of both *inlA* and *actA* may reflect specific mutations or deletions affecting host cell invasion and intracellular dissemination. Although the lack of these virulence determinants may influence pathogenic mechanisms, the ability of *L. monocytogenes* to cause disease cannot be inferred from single genes alone, but rather from the combined effect of multiple virulence and regulatory factors. As for other pathogenicity islands such as LIPI‐3 and LIPI‐4, they tend to be associated with specific hypervirulent CCs and are strongly linked to Lineage I isolates [69] and are less common among food isolates. Their absence in strains isolated in this study aligns with previous findings that food‐derived strains often lack these additional virulence islands. Taken together, these findings indicate that the virulence gene profile observed in this study reflects a predominance of food‐adapted Lineage II strains rather than highly invasive Lineage I clones.

Other valuable information regarding genetic diversity among RTE isolates from local city markets is provided through WGS data and SNP analysis. This study used SNP analysis to determine the genetic diversity between the 11 isolated strains. Previous studies described common source outbreaks and their diversities by using SNP analysis. Some studies suggest that for *L. monocytogenes,* a threshold of fewer than 20 SNPs indicates an epidemiological link between isolates [70]. Others suggest that the number of SNP differences between isolates within an outbreak of foodborne disease is considered to range from 0 to 12 SNPs [71–74]. However, there is some variation between studies regarding the classification of strains as closely linked. In the current study, the criteria described by Kwong et al. [71] were used for estimating the possible genomic relatedness of *L. monocytogenes* strains. Although these thresholds are primarily derived from outbreak investigations, their application in the context of food surveillance allows the identification of closely related strains that may indicate shared contamination sources or persistent clones within the food chain. In this study, closely related strains LM38 and LM43 originated from the same producer, differing by only seven SNPs, whereas LM24 and LM35 were also closely related (seven SNPs) but originated from different producers. For the latter, contamination may have been derived from a shared food source, suggesting a potential common source of contamination. The detection of such closely related strains among retail RTE products is of particular relevance, as it suggests potential contamination during production, processing, or distribution. Conversely, the high SNP distances observed among the remaining strains indicate substantial genetic diversity, supporting the presence of multiple, independent contamination events rather than a single point source. This finding further supports the heterogeneous population structure of *L. monocytogenes* circulating in retail RTE products in Croatian markets.

Based on the given data, for Croatia, studies on *L. monocytogenes* in food exist, but data on genotypic characteristics remain scarce. All samples in this study were traditional RTE meat products obtained from retail markets. Microbiological analyses were conducted according to EU Regulation No. 2073/2005 [14], and all samples met the regulatory limit of < 100 CFU/g at the time of analysis. Despite compliance with microbiological criteria, the detection of genetically related strains among retail RTE products indicates that low bacterial counts do not exclude the circulation of persistent and widely distributed clones within the food chain. Nonetheless, the presence of a hypervirulent CC1 strain and the detection of virulence genes in all isolates underline the potential public health risk posed by these products. According to the EFSA scientific opinion [46], the epidemiological and quantitative microbiological risk assessment data, the absence of *L. monocytogenes* in 25 g of food is required for susceptible populations and for foods that support the growth of the microorganism. Furthermore, public health risk exists with all disease‐causing organisms, and there is no “zero” risk for human illness. Epidemiological data from specific outbreak investigations suggest that low levels of *L. monocytogenes* may be associated with infection in susceptible populations [75]. However, the infectious dose depends on multiple factors, including host susceptibility, strain virulence, and the food matrix, and remains difficult to define [54, 76]. SNP‐based WGS analysis provided additional resolution beyond conventional genotyping methods, allowing discrimination between closely related and genetically unrelated strains isolated from similar food matrices. The identification of both closely related and genetically diverse strains suggests that contamination of RTE meat products likely originates from multiple independent sources rather than a single contamination event, highlighting the complexity of contamination routes in traditional food production. The findings of this study support the need for continued monitoring, genotypic surveillance, and characterization of *L. monocytogenes* strains, particularly in traditional RTE food products, to ensure consumer safety and prevent future outbreaks. In this context, SNP‐based analysis represents a valuable tool for food safety surveillance, as it enables early detection of potentially related strains and serves as a genomic baseline essential for future comparisons with clinical isolates and for the timely recognition of emerging clusters.

## 5. Conclusion

This study provides valuable data on the molecular serogrouping and genotyping of *L. monocytogenes* strains isolated from traditional dried meat products available in Zagreb, contributing to the epidemiological characterization of circulating strains in Croatian retail markets. The identified CCs reflect known pathogenic profiles: While hypervirulent clones such as CC1 and CC8 are more frequently associated with invasive human listeriosis, hypovirulent clones such as CC9, CC37, and CC121—predominant in this study—are mainly linked to infections in immunocompromised individuals. Despite this classification, all isolates harbored key virulence genes, including LIPI‐1 and internalins (*inlA* and *inlB*), emphasizing their potential to cause disease.

These findings highlight the potential public health risk associated with the presence of genetically diverse and, in some cases, closely related *L. monocytogenes* strains in traditional RTE meat products, and reinforce the need for targeted surveillance and molecular monitoring of this pathogen in such food matrices. Furthermore, SNP‐based WGS analysis provided higher resolution discrimination between associated strains, demonstrating the importance of WGS for identifying related isolates and supporting future risk‐based characterization of *L. monocytogenes*. Future studies should include whole‐genome comparisons of foodborne and clinical isolates to improve understanding of transmission dynamics and to support rapid outbreak detection and response. However, it should be noted that this study has certain limitations, including the relatively small number of samples analyzed, which may limit the representativeness of the findings at the national level. Despite these limitations, the results provide valuable baseline data on the occurrence and genetic diversity of *L. monocytogenes* in traditional RTE meat products and highlight the applicability of WGS‐based approaches for food safety surveillance.

## Funding

No funding was received for this manuscript.

## Conflicts of Interest

The authors declare no conflicts of interest.

## Supporting Information

Additional supporting information can be found online in the Supporting Information section.

## Supporting information


**Supporting Information 1** Table S1: Primer sequences and concentrations.

**Supporting Information 2** Table S2: Pairwise SNP distance matrix of the analyzed *Listeria monocytogenes* isolates.

**Supporting Information 3** Figure S1: Capillary gel electrophoresis gel image showing serotyping results for 11 *L. monocytogenes* strains.

## Data Availability

The data that support the findings of this study are available from the corresponding author upon reasonable request.
