# Supplementary material for: Listeria monocytogenes in Traditional Ready‐to‐Eat Dry Meat Products From Zagreb, Croatia: Occurrence and Genotyping
Source: Int J Microbiol. 2026 Jul 10;2026:1946018. doi: 10.1155/ijm/1946018 (PMC13351617; doi:10.1155/ijm/1946018)
Supplement: Supplementary file 1 — Supporting Information 1 Table S1: Primer sequences and concentrations. [file IJM-2026-1946018-s002.docx]

Table S1. Primer sequences and concentrations

| **Target gene** | **Primer sequence (5′-3′)** | \| **Product size (bp)** \|  \| \| --- \| --- \| | **concentrations** |
| --- | --- | --- | --- | --- | --- |
| lmo0737 1 | AGGGCTTCAAGGACTTACCC | 691 | 0.2 µM |
| lmo0737 2 | ACGATTTCTGCTTGCCATTC |  | 0.2 µM |
| lmo1118 1 | AGGGGTCTTAAATCCTGGAA | 906 | 0.4 µM |
| lmo1118 2 | CGGCTTGTTCGGCATACTTA |  | 0.4 µM |
| *ORF2819 1* | AGCAAAATGCCAAAACTCGT | 471 | 0.2 µM |
| *ORF2819 2* | CATCACTAAAGCCTCCCATTG |  | 0.2 µM |
| *ORF2110 1* | \| AGTGGACAATTGATTGGTGAA \|  \| \| --- \| --- \| | 597 | 0.2 µM |
| *ORF2110 2* | \| CATCCATCCCTTACTTTGGAC \|  \| \| --- \| --- \| |  | 0.2 µM |
| prs 1 | GCTGAAGAGATTGCGAAAGAAG | 370 | 0.2 µM |
| prs 2 | \| CAAAGAAACCTTGGATTTGCGG \|  \| \| --- \| --- \| |  | 0.2 µM |
