# Supplementary material for: Listeria monocytogenes in Traditional Ready‐to‐Eat Dry Meat Products From Zagreb, Croatia: Occurrence and Genotyping
Source: Int J Microbiol. 2026 Jul 10;2026:1946018. doi: 10.1155/ijm/1946018 (PMC13351617; doi:10.1155/ijm/1946018)
Supplement: Supplementary file 3 — Supporting Information 3 Figure S1: Capillary gel electrophoresis gel image showing serotyping results for 11 L. monocytogenes strains. [file IJM-2026-1946018-s001.docx]

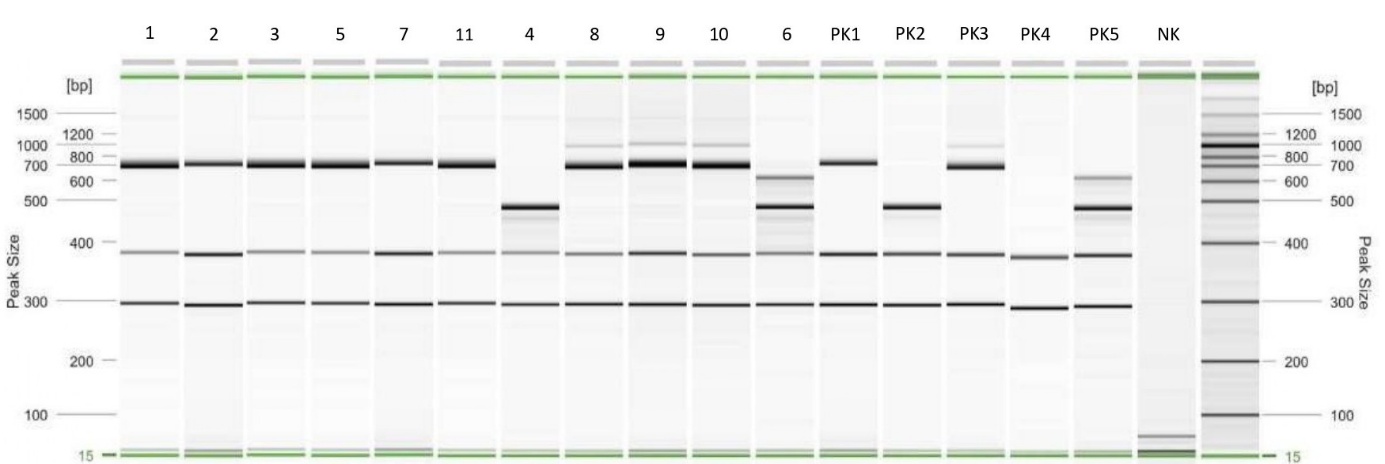


Figure S1. Capillary gel electrophoresis gel image showing serotyping results for 11 *L. monocytogenes* strains. On the left side of the image are the size marker and alignment markings (50-2500 bp size marker and 15-3000 bp alignment marker were used). Marks: PK1 – positive control 2a (serogroup IIa); PK2 – positive control 2b (serogroup IIb); PK3 - positive control 2c (serogroup IIc); PK4 – positive control 4a (serogroup IVa); PK5 – positive control 4b (serogroup IVb); NK – negative control
